# Microbiome of Acute Otitis Externa

**DOI:** 10.3390/jcm11237074

**Published:** 2022-11-29

**Authors:** Sung Kyun Kim, Sung Jun Han, Seok Jin Hong, Seok Min Hong

**Affiliations:** Department of Otorhinolaryngology-Head and Neck Surgery, Dongtan Sacred Heart Hospital, Hallym University College of Medicine, #7 Keunjaebong-gil, Hwaseong 18450, Gyeonggi-do, Republic of Korea

**Keywords:** microbiome, otitis externa

## Abstract

Background: Ear canal skin is directly attached to bone or cartilage, and is also connected to the eardrum. Acute otitis externa is cellulitis of the ear canal skin and subdermal tissue associated with acute inflammation and variable edema. We characterized the microbiome of the normal ear canal and ear canal with otitis externa. Methods: In total, 28 samples (14 each from the ear canal skin of patients with acute otitis externa and normal healthy controls) were collected using swabs. DNA extraction and bacterial microbiome analysis via 16S rRNA gene sequencing were performed. Results: The diversity index (mean amplicon sequence variants and Shannon index) were lower in the otitis externa than control group. According to linear discriminant effect size (LEfSe) analysis, a number of taxa differed significantly between the groups. *Pseudomonas* at the genus level and *Staphylococcus warneri* at the species level were identified in the otitis externa group. Conclusion: Our results show the importance of the microbiome in the pathogenesis of otitis externa and provide a basis for treating acute otitis externa by targeting the microbiome.

## 1. Introduction

The skin forms a protective barrier against environmental pathogens. Microorganisms colonize the skin surface, forming units that contribute to immunity. Unlike other types of exposed skin, ear canal skin is directly attached to bone or cartilage, and is also connected to the eardrum. Functionally, ear canal skin discharges tissue fragments falling from the eardrum and surrounding skin [1].

Acute otitis externa is a cellulitis of the ear canal skin and subdermal tissue associated with acute inflammation and variable edema. Most (98%) cases of acute otitis externa in North America are bacterial [2]. The most common pathogens are *Pseudomonas aeruginosa* (20–60% prevalence) and *Staphylococcus aureus* (10–70% prevalence), which occur as a polymicrobial infection [3].

Advances in next-generation sequencing (NGS) have given rise to metagenomic analysis, which typically targets the 16S ribosomal RNA (16S rRNA) gene and enables characterization of the complex microbial communities of the human body [4]. The microbiome plays roles in health and disease states [5,6,7] by contributing to immune responses and tissue repair. Disruption of the microbiome can result in inflammation or infection, potentially leading to various pathophysiological conditions and diseases [8] such as asthma [9], allergic rhinitis [10], atopic dermatitis [11], cardiovascular diseases [12], and neurodegenerative diseases [13]. Therefore, NGS can provide insight into the pathogenesis of acute otitis externa.

We characterized the microbiome of the normal ear canal and ear canal with otitis externa in this study.

## 2. Materials and Methods

### 2.1. Subjects and Clinical Variables

The study was approved by our Institutional Review Board (no. 2020-03-015), and all subjects provided written informed consent prior to the start of the study. The subjects were 14 patients with acute otitis externa and 14 normal healthy control. The diagnosis of acute otitis externa was confirmed if the patient had symptoms of ear canal inflammation such as otalgia, itching, or fullness, as well as ear canal edema, erythema, or otorrhea on ear endoscopy, and a rapid onset (<48 h). Normal healthy controls had no history of otitis externa. The exclusion criteria were a history of otitis media, age < 18 years, and use of oral antibiotics or ear drops containing antibiotics or steroid for ≥6 weeks.

### 2.2. Sample Collection and DNA Extraction

Swabbing was performed from the ear canal of patients with acute otitis externa, and the right or left side ear canal of normal healthy controls. To avoid contamination during swabbing, we used an Eswab (482CE; COPAN, Brescia, Italy) consisting of a minitip, and swabbing was conducted after carefully entering the tip of the swab into the ear canal without contacting the concha. Swabbing was performed by fully rotating the swab at least five times in the ear canal. After swabbing, the swab was placed in a collection tube and the remainder of the stem was discarded. Tubes were capped and transported to the laboratory for DNA extraction.

Swabs were vortex-mixed for 10 s to release organisms. Next, 1 mL of transfer buffer was transferred to a new tube, and remaining buffer was obtained by squeezing the swab. A bacterial pellet was generated by centrifugation for 10 min at 7500 rpm. Total DNA was extracted using the QIAamp DNA Mini Kit (QIAGEN, Hilden, Germany) according to the manufacturer’s instructions. DNA concentration and purity were measured using a UV-VIS Spectrophotometer (Quawell, Sunnyvale, CA, USA). Extracted DNA was stored at −70 °C until sequencing.

### 2.3. Library Construction and Sequencing

Sequencing libraries were prepared according to the Illumina 16S Metagenomic Sequencing Library protocols (Illumina, San Diego, CA, USA) to amplify the V3 and V4 regions. Input gDNA (2 ng) was PCR-amplified with 5× reaction buffer, 1 mM dNTP mix, 500 nM each of universal forward and reverse PCR primers, and Herculase II Fusion DNA polymerase (Agilent Technologies, Santa Clara, CA, USA). The conditions of the first PCR were 3 min at 95 °C for activation, and 25 cycles of 30 s at 95 °C, 30 s at 55 °C and 30 s at 72 °C, followed by a 5 min final extension at 72 °C. The universal primers and Illumina adapter overhang sequences for the first amplification were as follows:

16S Amplicon PCR forward primer 5′-TCGTCGGCAGCGTCAGATGTGTATAAGAGACAGCCTACGGGNGGCWGCAG-3′

16S Amplicon PCR reverse primer 5′-GTCTCGTGGGCTCGGAGATGTGTATAAGAGACAGGACTACHVGGGTATCTAATCC-3′

The first PCR product was purified using AMPure beads (Agencourt Bioscience, Beverly, MA, USA). Following purification, 2 µL of the first PCR product was PCR-amplified using Nextera XT Indexed Primer. The conditions were identical to the first PCR, except that 10 cycles were used. The PCR product was purified using AMPure beads and quantified by qPCR according to the qPCR Quantification Protocol Guide (KAPA Library Quantification kits for Illumina sequencing platforms), qualified using the TapeStation D1000 ScreenTape (Agilent Technologies, Waldbronn, Germany), and sequenced on the MiSeq™ platform (Illumina).

### 2.4. Bioinformatics Pipeline and Statistical Analysis

Raw Illumina MiSeq data were classified using an index sequence, and a paired-end FASTQ file was created for each sample. Using Cutadapt (v. 3.2) software, adapter and F/R primer sequences of the target gene region were removed. To correct errors in amplicon sequencing, the DADA2 (v. 1.18.0) package in R (v. 4.0.3) software was used. For paired-end reads, the forward sequence (Read 1) and reverse sequence (Read 2) were cut to 250 and 200 bp, respectively, and sequences with expected errors of ≥2 were excluded. Next, an error model for each batch was established to remove noise. After assembling the paired-end sequences into a single sequence, the chimera sequence was removed using the DADA2 consensus method to derive amplicon sequence variants (ASVs). For comparative analysis of the microbial community, the minimum number of reads among all samples was determined using QIIME (v. 1.9) software, and subsampling was then performed.

The Shannon index was calculated to assess microbial species diversity and uniformity, and alpha diversity information was verified by generating a rarefaction curve and calculating Chao1 values. Beta diversity was evaluated based on the weighted and unweighted UniFrac distances, and relationships between samples were visualized by principal coordinates analysis (PCoA).

The Wilcoxon rank-sum test was applied to confirm the difference in alpha diversity between the groups, and FDR correction was performed using the Benjamini–Hochberg method to adjust for multiple testing. For visualization of the results of between-group comparisons, the ggplot (v. 3.2.1) package in R (v. 4.0.3) software was used. Linear discriminant effect size (LEfSe) analysis was performed to compare the microbial community composition between groups. Microorganisms showing a significant difference were selected and the degree of difference was expressed as an LDA Score.

## 3. Results

### 3.1. Demographic Data

A total of 28 subjects (14 patients with otitis externa and 14 normal healthy controls) were recruited to this study. In the otitis externa group, there were eight males and six females, and the average age was 41.8 years (range: 23–69 years). In the normal healthy control group, there were six males and eight females, and the average age was 48.1 years (range: 40–63 years). The two groups were matched in terms of sex and age.

### 3.2. Bacterial Diversity and Community Composition

**Figure 1:**
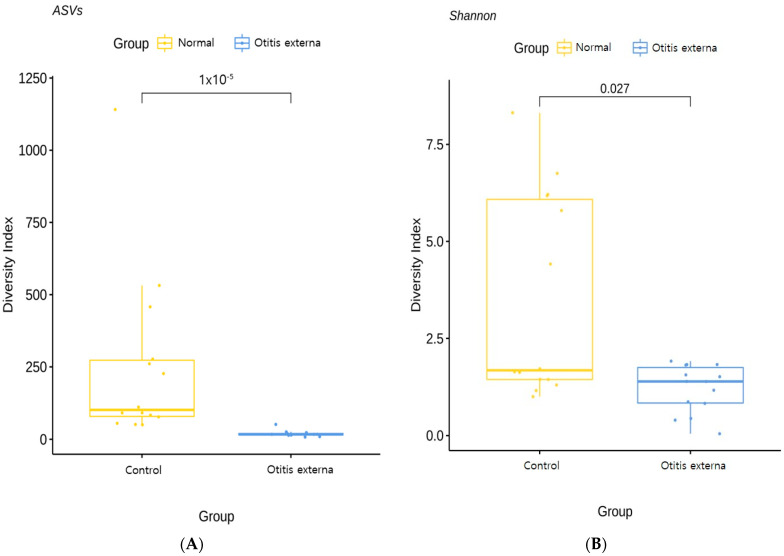
Box plot of the ASV-based analysis (**A**) and Shannon index values (**B**) for diversity. Boxes represent second or third quartiles. ASV (amplicon sequence variant): inferred single DNA sequences recovered from a high-throughput analysis of marker genes.

The diversity index of the otitis externa group was significantly lower than that of the healthy control group. The mean ASV was 19.4 in the otitis externa group and 250.4 in the control group. The mean Shannon index was 1.21 in the otitis externa group and 3.49 in the control group, indicating that the otitis externa group had lower bacterial diversity than the control group (Figure 1).

**Figure 2:**
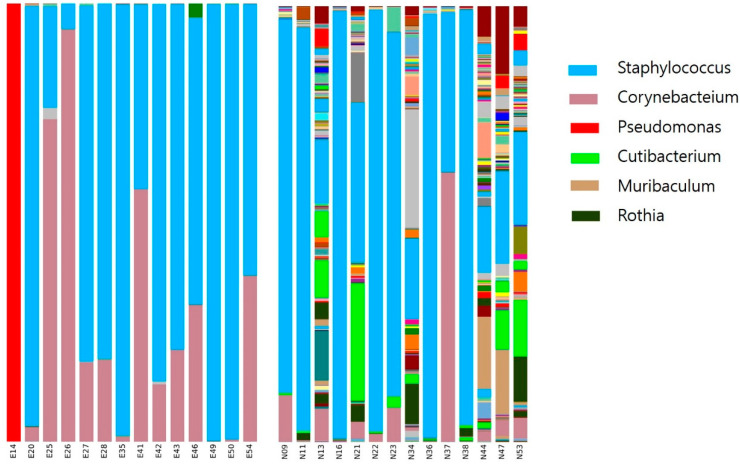
Taxonomic analysis at the genus level. E, external otitis; N, normal healthy control; number, sample number.

At the genus level, microbial diversity was higher in the control than otitis externa group. In the control group, *Staphylococcus*, *Cutibacterium*, *Corynebacterium*, *Pseudomonas*, *Muribaculum*, and *Rothia* were detected, among other taxa. In the otitis externa group, *Staphylococcus*, *Corynebacterium*, and *Pseudomonas* accounted for the majority of the bacterial community (Figure 2).

**Figure 3:**
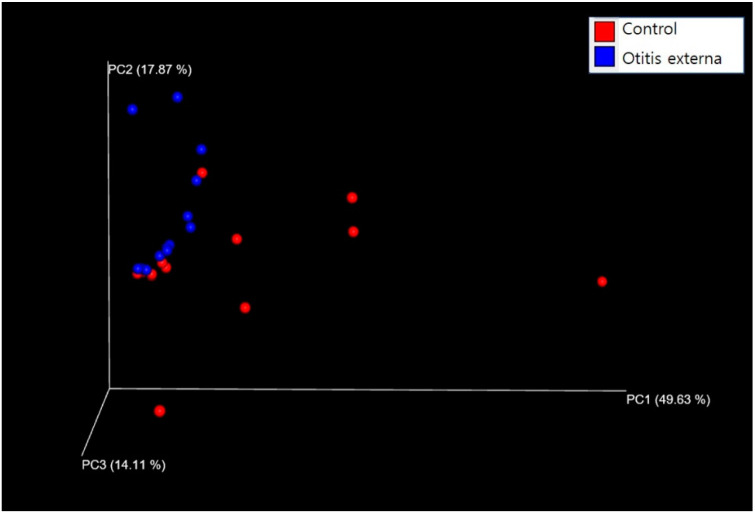
Three-dimensional principal coordinates analysis based on the weighted UniFrac distance matrix. Colored spheres represent samples. The percentages for PCs 1–3 denote the variance explained by each PC.

The diversity and associations of bacterial composition between the otitis externa and control groups were determined using PCoA, which can distinguish between groups of samples in the coordinate plane. The two groups appeared to be separated based on principal component (PC) 1 axis, but did not constitute completely separate clusters (Figure 3).

**Figure 4:**
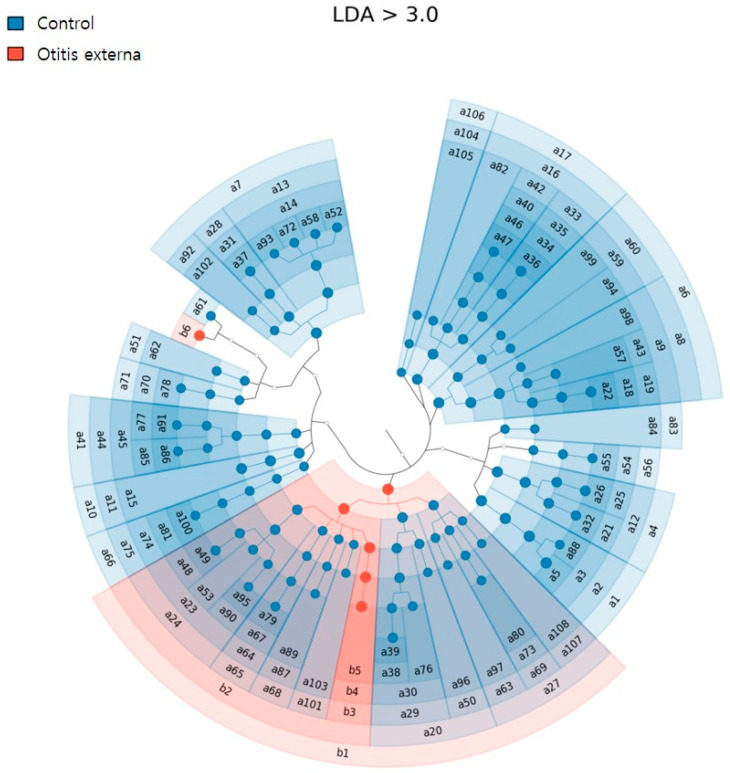
Results of Linear discriminant analysis effect size (LEfSe) analysis. Regions in red and blue indicate taxa significantly enriched in the otitis externa and control groups, respectively. Each ring represents a taxonomic level, with phylum in the outermost ring and species in the innermost ring. a: distinct taxa in control group, b: distinct taxa in otitis externa group. The number indicates an individual of taxa at each taxonomic level.

To identify species that could differentiate the groups, linear discriminant analysis effect size (LEfSe) analysis was conducted. At a cut-off value of LDA of ≥3, 114 significantly distinct taxa were found (6 in the otitis externa group and 108 in the control group). Among them, *Pseudomonas* (genus) and *Staphylococcus warneri* (species) were identified in the otitis externa group. In the control group, 25 genera (e.g., *Cutibacterium*, *Streptococcus*, *Muribaculum*, and *Rothia*) and 17 species (e.g., *Cutibacterium acne, Muribaculum intestinale, Micrococcus aloeverae,* and *Rothia mucilaginosa*) were identified (Figure 4).

## 4. Discussion

Acute otitis externa is an infection of the skin of the outer ear canal that typically presents as otalgia, with or without ear discharge. It is a common condition with an annual incidence of 1% that affects 10% of the general population during their lifetime [14]. *P. aeruginosa* and *S. aureus* are common pathogens in otitis externa [3]. However, previous studies used bacterial culture methods; therefore, some unculturable bacteria were likely to have been underestimated, whereas other bacteria with high proliferation rates might have been overestimated [15].

New techniques, such as 16S and whole-genome shotgun sequencing, have provided insight into the diversity and functions of the skin microbiome [16]. The skin microbiome composition is dependent on the physiology of the skin site, and specific bacterial taxa inhabit dry, moist, and sebaceous microenvironments [17]. Sebaceous skin sites are dominated by *Propionibacterium* and *Staphylococcus*, whereas *Corynebacterium* prefers moist sites [16]. Ear canal skin apocrine (ceruminous) and sebaceous glands have been analyzed [18], but there are few studies on the microbiome of the human ear canal [19]. In a culture study, *Staphylococcus* was the most abundant taxon, followed by *Corynebacterium* and *Streptococcus* [20]. In this study, *Pseudomonas*, *Muribaculum*, and *Rothia* were detected in the control group, in addition to *Staphylococcus*, *Cutibacterium*, and *Corynebacterium*.

In this study, the alpha-diversity of the microbiome such as ASV or Shannon index in the otitis externa group was significantly lower compared to the control group. Additionally, also beta-diversity such as PCoA indicated differences in microbial composition between the otitis externa and control groups. In a study, alpha diversity did not differ significantly between the otitis externa and healthy control groups [21]. However, in several previous studies, decreased microbiome diversity was reported in inflammatory and infectious diseases [22,23], and changes in microbiome composition compared to normal skin were reported in skin diseases such as atopic dermatitis [22,23,24]. Innate immunity is believed to be influenced by the microbiome composition via the effects of the microbiota on the entire physiology of the host organism; the microbiota influences multiple facets of homeostasis via its effects on the innate immune system [25]. Additionally, the microbiome reinforces the epithelial barrier, induces the expression of regulatory T cells, and interacts with the innate immune system [26].

Skin commensals maintain epithelial homeostasis and tissue health [17]. The equilibrium of pathogenic and non-pathogenic strains normally ensures that commensals remain benign or beneficial; however, a shift in microbial composition may provide space for the growth of harmful species. Consequently, newly dominant strains can trigger skin inflammation and diseases. Opportunistic pathogens adhere to skin, disrupt the epithelial barrier, and trigger skin infection or inflammation [8,27].

LEfSe finds data (taxon) that show a significant difference between comparison groups using LDA score, and is an analysis method that expresses how much difference the found taxon shows between groups on a log scale. The higher the LDA, the larger the difference between groups and the smaller the variance within groups. That is, a high LDA score do not mean that it is commonly observed in the group, but means that the data show a clear difference between groups. Indeed, in this study, *Staphylococcus aureus* was most commonly detected in the otitis externa, but the species with the highest LDA score was *Staphylococcus warneri*. Additionally, many other distinct taxa were found in the LEfSe analysis.

In the control group, there were significantly more taxa than in the otitis externa group. This is likely because, even if a fairly small amount of taxon was detected in control group, if it was not found in the otitis externa group, it has been analyzed as a distinct taxon. In the otitis externa group, *Pseudomonas* (genus) was identified as a distinct taxon. Of the pseudomonads, *Pseudomonas aeruginosa* is known to be the most common causative pathogen of acute external otitis. This opportunistic pathogen causes a range of infections, is present in many environments, and is often part of the human skin microbiome. However, the mechanism by which *P. aeruginosa* establishes infection in the ear canal is unclear [28]. *Staphylococcus warneri*, a distinct taxon at the species level, is a catalase-positive, oxidase- and coagulase-negative skin commensal that constitutes <1% of the skin *Staphylococcus* population [29]. Although it is a rare cause of infection, *Staphylococcus warneri* was the sixth most frequently isolated *Staphylococcus* species in acute otitis externa [21]. Therefore, the commensals *Pseudomonas* and *Staphylococcus warneri,* as pathogens in the ear canal, may affect the epithelial barrier and innate immunity, play a role as pathogens under the environment of changes in the composition of the microbiome of the ear canal. Recently, oral probiotics indirectly influence skin diseases, and a number of topical probiotic formulations have been proposed to ameliorate skin conditions by suppressing inflammation and restoring the skin microbiome balance [30]. Additionally, the findings of this study were expected to be facilitate treatments for acute otitis externa targeting the microbiome.

Our microbiome results have several differences from previous reports [19,31]. Microbiome composition is affected by many factors, such as the host genome, age, sex, family history, nutrition, and hormones. Other qualities of the external auditory canal), such as wet/dry cerumen type, can also affect microbiome. In East Asian populations, dry-type cerumen is prevalent, whereas the wet type is more common in Europeans and Africans [31]. These factors may explain the differences in results between our and previous studies.

There are limitations to this study. First, the sample size is small. Additionally, it is difficult to generalize any theory as a result of this study. Since our hospital is a secondary hospital, many patients are referred from primary hospitals. Therefore, it was difficult to enroll patients who had not used antibiotics for the last 6 weeks. Further studies with larger samples are needed in the future. Second, sampling was not performed in the contralateral ear of patients with acute otitis externa. Patients with otitis externa may have a habit of frequently picking their ears with a swab. Therefore, since this habit can affect the microbiome of the ear canal, in this study, people without a history of otitis externa were used as a control group. However, if the contralateral ear canal is normal, comparing the microbiome of the affected side and the contralateral ear canal of one person under the same environment may have shown better results in identifying the role of the microbiome in the development of otitis externa.

## 5. Conclusions

Bacterial diversity and microbial composition differed between our otitis externa and normal healthy control groups and total 114 significantly distinct taxa were found (6 in the otitis externa group and 108 in the control group). Our results highlight the importance of the microbiome in the pathogenesis of acute otitis externa. In particular, based on the distinct strains of LEfSe analysis, further studies on functional gene analysis, their metabolites, secreted peptides, and interactions between humans and microbiome are needed. Additionally, we suggest that it could be targeted for treatment and prevention of the condition.

## Data Availability

The data used to support the findings of this study are available from the corresponding author upon request.
